# Positional Quality Assessment of Orthophotos Obtained from Sensors Onboard Multi-Rotor UAV Platforms

**DOI:** 10.3390/s141222394

**Published:** 2014-11-26

**Authors:** Francisco Javier Mesas-Carrascosa, Inmaculada Clavero Rumbao, Juan Alberto Barrera Berrocal, Alfonso García-Ferrer Porras

**Affiliations:** 1 Department of Graphic Engineering and Geomatics, University of Cordoba, Campus de Rabanales, 14071 Cordoba, Spain; E-Mails: inmaclavero@uco.es (I.C.R.); agferrer@uco.es (A.G.-F.P.); 2 Departamento de Suelos y Recursos Naturales Facultad de Agronomía, Universidad de Concepción, 3780000 Concepción, Chile; E-Mail: jbarrera@udec.cl

**Keywords:** UAV, positional quality, orthophoto

## Abstract

In this study we explored the positional quality of orthophotos obtained by an unmanned aerial vehicle (UAV). A multi-rotor UAV was used to obtain images using a vertically mounted digital camera. The flight was processed taking into account the photogrammetry workflow: perform the aerial triangulation, generate a digital surface model, orthorectify individual images and finally obtain a mosaic image or final orthophoto. The UAV orthophotos were assessed with various spatial quality tests used by national mapping agencies (NMAs). Results showed that the orthophotos satisfactorily passed the spatial quality tests and are therefore a useful tool for NMAs in their production flowchart.

## 1. Introduction

The widespread and growing use of geographic data has led to a high demand for this information, which is reflected in every aspect of our daily life. Technological progress has contributed to “democratize” the cartographic communication processes. Users demand more and more information and governments require good quality data. In the United States, the Federal Geographic Data Committee [1] reported that 80% to 90% of government information has a geospatial component. To be useful, geographic information requires accuracy in all its components (*i.e.*, spatial, temporal, topological and thematic). In this context, images acquired by unmanned aerial vehicle (UAV) platforms are very useful because of their high spatial and temporal resolution. This makes them interesting for national mapping agencies (NMAs) in their flowchart of geographic data production. Such UAV products are considered useful as long as they meet the technical requirements of NMAs.

One of the most important quality features of cartographic products is positional accuracy, which is the key for interoperability between geodatabases [2] and is evaluated by NMAs [3]. Given that positional quality is essential in cartographic production, all NMAs use statistical methods to control it [4]. Producers or users can assess the spatial quality of cartographic products using several tests and standards such as the NMAS, STANAG or NSSDA [5–7]. The spatial accuracy of UAV orthophotos is usually reported using the root mean square error (RMSE) [8–10]. However, it is also interesting to assess the quality of such orthophotos according to standard tests used by NMAs. Cramer *et al.* [11] provided a state of the art overview on the use of UAV by some European NMAs. They summarize that NMAs are following the most recent developments and are working on a possible integration of UAV in their production lines.

The scenario where geographic data are used has changed in the last decade. Users have easier access to geospatial data and producers have a wide range of platforms and sensors to obtain data with. These changes represent progress as long as users and producers make a good use of geographic information. As regards the quality of data, the ISO 9000 standard defines quality as “the totality of characteristics of an entity that bear on its ability to satisfy stated and implied need”. Juran *et al.* [12] defined quality as “fitness for use”. Quality can be understood as the closeness of the agreement between data characteristics and explicit or implicit needs of a user for a given application [13]. Therefore, a cartographic product may be useful or not depending of the use that is made of it. It is important to assess the spatial, spectral, radiometric and temporal resolution of the product. Because of this, it is important to know the objectives of a project to decide which platforms, tools and sensors to use. Thus, in cartography obtained with UAVs, as in that obtained with other methodologies or platforms, it is important to assess the quality of all its components, including spatial resolution.

Geographic data can be obtained through different means, ranging from airborne to field devices. Aerial means to obtain data include a broad range of sensors that can be used in three main types of platforms: satellite, manned aerial or UAV platforms. Depending on the intended use of data provided by onboard sensors, such platforms may or may not be adequate because of their different resolutions. Satellite-based products have a limited application to some projects because they have low spatial resolution [14]. Conversely, very high spatial resolution images obtained by metric cameras on board aircraft tend to have low spectral resolution. In addition, both aerial and satellite platforms share the problem of having limited temporal resolution. Many applications such as precision farming, fire monitoring or civil engineering require timely availability of data and sometimes even real-time data. In these scenarios, conventional satellite and manned aerial platforms are not adequate because of their low temporal resolution under normal conditions. UAVs provide an alternative to such platforms. NMAs can use UAVs as a tool to update geodatabases, improving the temporal resolution of their products.

The methods available to obtain data or to produce geomatic products are progressing very fast and there is a great need for updated cartographic products because such products rapidly become obsolete [15]. This has led to a great interest in using UAVs not only for military purposes [16], but also for civil applications [17]. UAV platforms can also be used in scientific, public safety and commercial tasks such as data and image acquisition of disaster areas [18], map building [19], search and rescue operations [20], traffic surveillance [21], archaeology [22,23] and increasingly in forestry and agriculture [24].

Recent developments in modern navigation have led to the availability of reliable UAV platforms for photogrammetric surveys. Such platforms are not only used for surveillance or to obtain individual images but also to produce geomatic products such as digital surface models or orthophotos [25,26].

The benefits of using UAV platforms over traditional aircraft systems are related to mobilization costs, flexibility, number of operational flying days and very high spatial resolution data [27]. Such platforms can also be used in high-risk situations without endangering human lives, in inaccessible areas, at low altitudes and with flight profiles close to objects, where manned systems cannot be flown [28]. The advantages of territorial information obtained with UAVs are its lower cost compared to that obtained with conventional flights and the fact that high temporal and spatial resolution can be obtained when needed [8].

Moreover, advances in processing of data and measurement collected in UAV flights have been the key in the development of UAV services. These advances include calibration and aerial triangulation to produce digital models and orthophotos. Photogrammetric processing of UAV-based images have been described in several research projects [29,30]. Regarding calibration, three options are possible [31]. The first is to calibrate before the bundle adjustment, this option is used in [32]. The second option is to apply self-calibration described in [33]. The combination of both would be the third option reported in [34] and suggested in [35]. Referring to aerial triangulation, proven and traditional software has shown difficulties to process UAV blocks [36]. New softwares have been developed in consideration of characteristics of UAV blocks. Algorithms used in computer vision for general purposes like SfM [37], SIFT [38] and its variations like LDHahh [39] are used by photogrammetric UAV suites. In relation to surface reconstruction, Harwin and Lucieer [40] presents a compilation about techniques and their results for UAV point cloud generation.

The overall objective of the present research was to evaluate spatial accuracy in an orthophoto obtained by a multi-rotor UAV taking into account various standard tests used by NMAs. This paper is organized as follows: the materials and methods are described in Section 2, the results and discussion are addressed in Section 3 and the conclusions are presented in Section 4.

## 2. Results and Discussion

### 2.1. Study Site Description and Airborne Campaigns

**Figure 1.**
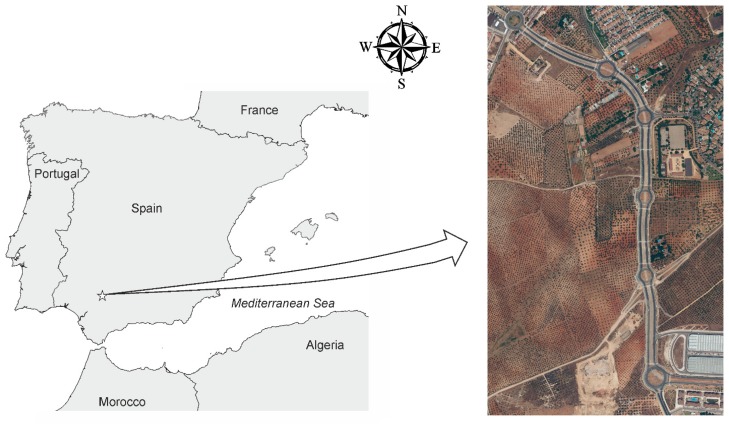
Overview of the study site.

The study was performed in Mairena del Aljarafe, in Seville province, southern Spain (37°21′N, 6°04′W). The area comprised a road with a soft slope and a length of 1.6 km between two urban areas. It included many planimetric details of interest to assess the spatial accuracy of the UAV orthophoto (Figure 1).

The unmanned aerial vehicle used for mapping was a MD4-1000 multi-rotor drone (Microdrones GmbH, Siegen, Germany). This UAV is a quadcopter with an entirely carbon design. It has a flying time of about 30 min using a 250 g sensor and an operation range of 500 m with radio control. The system has a maximum payload of 1.2 kg. It uses 4 × 250 W gearless brushless motors and reaches a cruising speed of 15.0 m/s. It can operate from a few meters to a ceiling altitude of 1000 m. The UAV was equipped with a Sony NEX-7 RGB sensor. This sensor provides an image of 23.5 × 15.6 mm, a focal length of 16 mm and an image size of 6000 × 4000 pixels. Its weight is 353 g including the camera body, card and battery.

The UAV was flown at an altitude of 200 m above ground level. Ground sample distance (GSD) was 5 cm, taking into account the characteristics of the sensor. The flight had a single strip configuration with two flight lines and an along-track overlap of 70%, providing 43 images. The flight was conducted in May 2013; it had a duration of 25 min and 54 s and a total length of 4.9 km.

#### GPS Campaign

The GPS campaign had two different objectives: (1) measure ground control points (GCPs) to use in the aerial triangulation phase; and (2) measure check points to assess the spatial accuracy of the resulting orthophoto. Data collection and observation were planned according to the manual of the U.S. Army Corps of Engineers [41]. The uncertainty of the check point coordinates had to be at least three times better than that of the target validation results [6]. To reach the maximum accuracy in the positioning we used two receivers: a reference station of the GNSS RAP network of the Institute of Statistics and Cartography of Andalusia, Spain, and a Leica GS15 GNSS rover receiver. For a final orthophoto with a scale of 1:500, the circular error is 0.2 m. Taking into account a Gauss distribution with a 95% probability, the root mean square error is 0.041 m. Since the source of higher accuracy (*i.e.*, check points) must be three times more accurate than the product, the mean square error must be 0.015 m. This proves that check point measures taken with this GNSS equipment are adequate.

**Figure 2.**
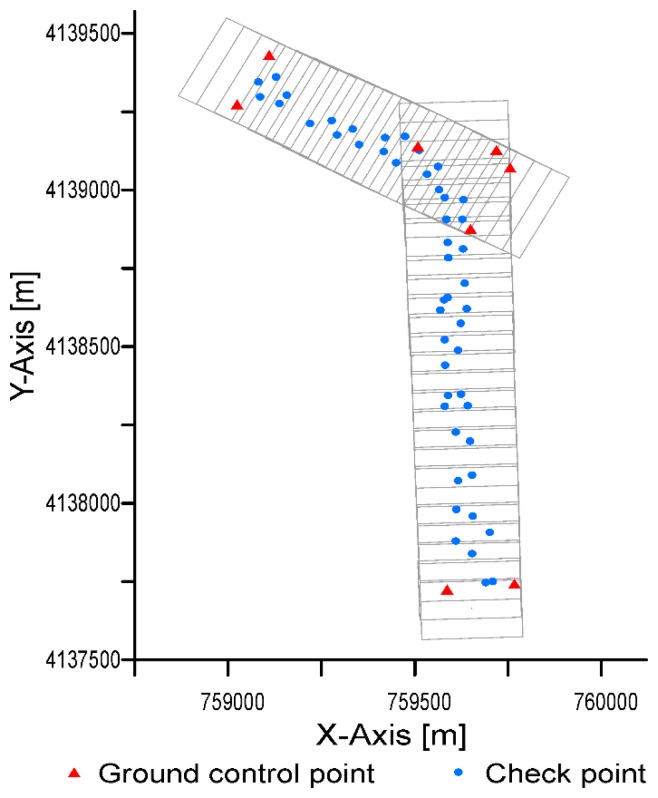
Distribution of ground control points and check points.

We used rapid static positioning to obtain the GCPs for use in aerial triangulation. Baseline length was about 15 km. For each point, the observation time was 15 min, with an update rate of one-second intervals. We measured a total of eight GCPs, located on the corners of each strip (Figure 2). We used fifty check points to assess spatial accuracy, measured with the Stop & Go technique for relative positioning with GPS, applying the Networked Transport of RTCM via Internet Protocol (NTRIP). The check points had a random and well-spaced spatial distribution (Figure 2). Twenty percent of the points were located in each quadrant of the working area, and the distance between points was at least 10% of the diagonal distance across the rectangle that enclosed the working area. All the check points were well-defined points in order to clearly identify them over the orthophoto. Check points were used in an external direct quality evaluation using different tests described below, in Section 2.3.

### 2.2. Photogrammetric Processing

Processing was divided into three stages. First, we performed the aerial triangulation. Next, we obtained a digital surface model (DSM). Both results made it possible to obtain individual orthorectified images and finally a mosaic image. The aerial triangulation and mosaic image were achieved using EnsoMOSAIC software (Mosaic Mill Ltd., Vantaa, Finland); the DSM was created using Rapid Terrain software (PIEneering Ltd., Helsinki, Finland).

The sensor calibration was performed using RapidCal software (Mosaic Mill Ltd.) before the flight, taking images of a calibration panel. Next, we adjusted the internal parameters of the sensor (*i.e.*, focal length, principal point and distortion coefficients).

In a previous stage, pyramid images were generated to optimize the image display and calculations. Currently at present, most UAS softwares apply fully automated procedures. However, sometimes it is necessary to measure points manually to improve the results in local areas of the photogrammetric blocks. Taking these circumstances into account we introduced some manual processing with the understanding that they could generate worse results. The images were manually linked to each other to determine the initial orientation of the images in relation to one another. This was achieved by identifying three visible objects in groups of two images, trying to define a triangle as open as possible. After that, we performed automatic aerial triangulation. Automatic tie points were located and used to perform a block adjustment. Three different strategies (*i.e.*, initial, intermediate and final) were used to search for tie points depending on the pyramid level image processed. In the initial strategy, we took into account in-flight collected GPS observations. In the intermediate strategy, we also used in-flight collected orientations. In the last strategy, we transferred the results obtained in the previous stage to the lowest level in the pyramid of images, that is, to the image with the highest spatial resolution.

After that, a DSM was generated using aerial triangulation data calculated in an earlier step. Every single image was orthorectified based on external orientations and the DSM. Finally, individual orthorectified images were mosaicked to obtain the UAV orthophoto of the area of interest. The GSD of the orthophoto was 0.05 m, the same as the GSD of the flight. We did not resample the orthophoto to a higher GSD because our goal was to determine the best scale at which the product can be used.

### 2.3. Spatial Accuracy Assessment

In this section we describe the various positional accuracy assessment methodologies (PAAMs) we applied to assess the orthophoto generated using the UAV platform. As a general rule, all the PAAMs were based on the assumption of errors with a normal distribution. They all consisted of statistical and testing methods to estimate the positional accuracy of points in digital geospatial data (*i.e.*, an orthophoto) using georeferenced check points with higher spatial accuracy referred to a coordinate reference system. Given that each PAAM assessed positional quality with different means, we used several PAAMs to analyze whether results were similar or not. The PAAMs used were the NMAS, EMAS, ASLSM, NSSDA and STANAG 2215.

From 1941 to the mid-1990s, most public and private sector cartographic organizations accepted the test National Map Accuracy Standards (NMAS) as an industry standard for large and small scale photogrammetric mapping. This test was developed in 1941 by the U.S. Bureau of the Budget [5]. It establishes a percentage of points that must not exceed a certain error. Only products that meet such conditions pass the test. In this study it was decided that a maximum of 10% of total check points could have an error greater than 0.850 m.

The second test we applied was the Engineering Map Accuracy Standard (EMAS), developed by the American Society of Civil Engineers [42]. This test is conducted in two stages. In the first stage, a t-Student test is used to assess the presence of bias. To pass the test, each individual coordinate component must meet |*t_x_*|≤*t_n_*_–1,_*_α_* and |*t_y_*|≤*t_n_*_–1,_*_α_*, where *t_n_*_–1,_*_α_* is the t-Student value of for n-1 degrees of freedom and a confidence level of *α*. *t_x_* and *t_y_* are equal to the statistic $t=\bar{e}\cdot \sqrt{n}/S$; *ē* is the mean error for each coordinate component, *n* is the number of points used in the test and *S* is the standard deviation. In the second step of the EMAS, the Chi-square test is used to determine whether random errors are adequate. It has to be verified that $\chi_{x}^{2}\leq \chi_{n-1,\alpha }^{2}$ and $\chi_{y}^{2}\leq \chi_{n-1,\alpha }^{2}$, where $\chi_{n-1,\alpha }^{2}$ is the value of Chi-square distribution for n−1 freedom degrees and a confidence level of *α*. $\chi_{x}^{2}$ and $\chi_{y}^{2}$ are equal to the statistic *χ*^2^ = *S*^2^ ·(*n*–1)/*a*^2^; *S* is the standard deviation, *n* is the number of points used and *a* is the maximum expected variance. This evaluation must be passed by both coordinate components simultaneously. Finally, the product is accepted if the results of both tests are satisfactory, so this methodology is more restrictive than the previous test.

The third methodology we used was the Accuracy Standards for Large-Scale Maps (ASLSM), developed by the American Society for Photogrammetry and Remote Sensing [43]. This methodology uses the root mean square error (RMSE) for each coordinate to check the product. Depending of the spatial accuracy of the product, the ASLSM defines three classes or types of products. The class of a product is assigned taking into account how the product has been compiled; the highest spatial accuracy class corresponds to C1, and C2 and C3 are two and three times worse, respectively. The standard defines a RMSE limit for each class depending of the scale. For a 1:500 scale, the RMSE limit is 0.125 m for C1, 0.250 m for C2 and 0.375 m for C3.

The fourth methodology was the National Standard for Spatial Data Accuracy (NSSDA), developed by the Federal Geographic Data Committee [6]. Instead of defining a threshold accuracy value, the NSDDA is a data usability standard in which agencies are encouraged to establish thresholds for their products and users have to determine the acceptable accuracy for their applications. This standard is mandatory for NMAs that produce maps in the United States. The RMSE is calculated for each component coordinate. If the RMSE for coordinate X is equal to the RMSE for coordinate Y, then the value of the NSDDA is calculated as *NSSDA* = 2.4477 × *RMSE_X_*. By contrast, if the RMSE is different for each component coordinate and 0.6 < (*RMSE_X_* + *RMSE_Y_*) < 1, then *NSSDA* = 2.4477 × 0.5 × (*RMSE_X_* + *RMSE_Y_*).

Finally, the North Atlantic Treatment Organization (NATO) developed Standardization Agreement 2215 [7], also known as STANAG. STANAG defines different accuracy ratings for absolute geometric accuracy. Each rate is identified by a letter from A to E, and A is considered the best accuracy rate. For each rate, it defines the maximum differences between any two well-defined points in map units. For a given scale it is possible to calculate the maximum measurement error. The test is conducted by calculating the circular map accuracy standard (CMAS) taking into account the circular standard deviation (*σ*_c_) as *CMAC* = 2.146*σ*_c_. The CMAS is compared to the maximum measurement error for a given rate and scale and must be lower than such error. In this case, the product is considered to be valid for a given scale and accuracy rate.

We took into account the different scales used in the tests describe above. Each scale was associated with a specific spatial requirement. Our intention was to determine the highest scale at which we could use our UAV orthophoto.

## 3. Results and Discussion

**Figure 3.**
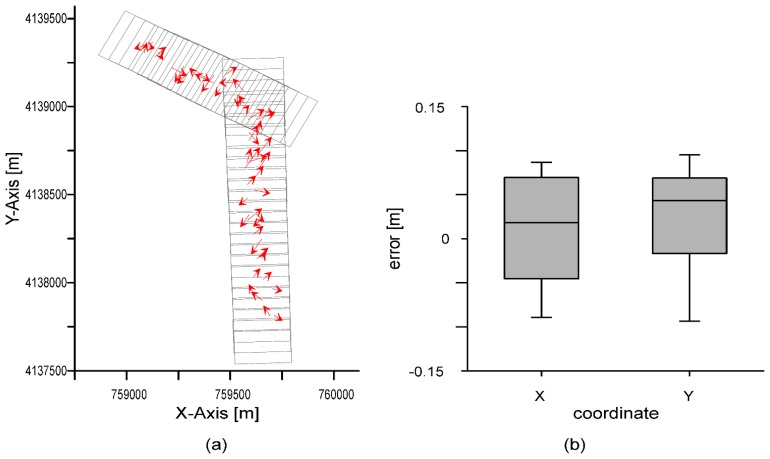
Comparison check points from GPS and UAV orthophoto: (**a**) distribution vector error; (**b**) box-plot graph of errors in coordinates X and Y.

The results of the five methods analyzed are presented in this section. The comparison between the coordinates of points extracted from the UAV orthophoto and their counterparts obtained with the GNSS receiver (*i.e.*, check points) showed a mean error of 0.012 and 0.022 m and a RMSE of 0.058 and 0.056 m for coordinates X and Y, respectively. Therefore, both coordinate components had errors in the same interval. Figure 3a shows the distribution of vector errors for each point. A priori, the spatial distribution of errors showed a random behavior, with no trends in the direction or orientation of the vectors. The magnitude of errors was also similar. Figure 3b shows a box-plot graph of the errors in each coordinate. Maximum and minimum errors in the X and Y axes were similar and did not exceed 0.10 m. This value amounts to twice the GSD of the UAV flight. In a previous stage, we noted that the orthophoto did not have outliers and all the errors passed the interquartile range rule, so all the measurements were used in the various tests we applied.

Table 1 summarizes the highest valid scale at which the UAV orthophoto can be used according to each test applied. It should be noted that there were a broad range of scales depending on the test used. Results of the NMAS test were optimistic. For a 1:250 scale, the percentage of points whose error was greater than 0.213 m (0.085 cm at that scale) was 0%. Taking into account that the human limit of visual perception is 0.2 mm, for a 1:250 scale such limit would be 5 cm, the same value as the GSD of the flight. This situation can be considered anomalous: in the cartographic process each stage contributes to increasing the error so it is not possible to produce an orthophoto where the GSD of the flight is the same as the GSD of the final product. Moreover, the manual measurement uncertainty of a coordinate point is at least the same as the GSD. Therefore, this test manifests a tolerant behavior because the defined tolerance is quite high, and the consequence is that producer error is almost zero.

The second test analyzed was the EMAS. In the first stage (*i.e.*, a systematic test), the value of t-Student's test considering a confidence interval of 95% and 49 degrees of freedom was 2.009. Coordinates X and Y passed this test with a value of 1.479 and 1.845 respectively. Therefore, there were no systematic errors in the orthophoto. In the second stage, the reference value for the Chi-square test considering a confidence interval of 95% and 49 degrees of freedom was 66.338. For a 1:1000 scale, the maximum expected variance (*a*) was 0.082 m. Therefore, the Chi-square value for the X and Y coordinates was 24.9 and 23.1, respectively. The 1:1000 scale was the highest that met the Chi-square test. When we analyzed a higher scale such as 1:500, the test did not pass the Chi-square test for coordinates X and Y. Results showed that this test was more restrictive than the NMAS because it required passing different tests at the same time for the X and Y components.

Taking into account the ASLSM test and considering a 1:500 scale and a Class 1 orthophoto, the standard establishes a value of 0.125 m as a planimetric coordinate accuracy requirement. The standard deviation for each coordinate component, shown at the beginning of this section, was lower than this limit. Therefore, the orthophoto was suitable for use at a 1:500 scale considering this standard.

The NSSDA test showed a result of 0.144 m. For a 1:500 scale, this value can be considered inadequate because this result is higher than the maximum error expected for this scale. It is more advisable to consider a 1:1000 scale to use the orthophoto. However, the result of the test should be displayed in the metadata of the product so that end users can decide how to use the data.

Finally, we applied the STANAG test. The circular map accuracy standard for 49 degrees of freedom was 0.134 m. Considering a measurement error of 0.5 mm for rate A and a 1:500 scale, the limit was 0.25 m, so the product reached rate A for this scale.

Summarizing the results of each test (Table 1), the tests covered a broad range of scales, from 1:250 to 1:1000. For a given scale it is possible to calculate the maximum error if we consider the visual perception limit of humans to be 0.2 mm. Under normal conditions, the GSD of the orthophoto is usually half of this limit. For a 1:250 scale, such limit is 0.050 m. The GSD orthophoto for this scale is 0.025 m. This value was lower than the GSD of the orthophoto so the result of the NMAS test was ruled out. Taking into account 1:500 and 1:1000 scales, the perception limits were 0.1 m and 0.2 m for each scale, respectively. Adequate GSD values for the orthophoto taking into account the limits of 1:500 and 1:1000 scales were 0.05 m and 0.10 m, respectively. In general, the GSD of the flight was lower than the GSD of the final orthophoto. Therefore, it would not be adequate to consider a 1:500 scale as valid because it is necessary to have a margin to take the errors of the process into account. In this case, considering a 1:500 scale, the GSD of the flight was equal to the GSD of orthophoto, which did not meet the requirements. Therefore, the UAV orthophoto would be adequate to use at a 1:1000 scale.

All the tests used are focused on the spatial resolution of the final product. We applied external direct quality evaluation to check it. These mapping standards do not cover other aspects related with spatial quality. A sample of ground control points probably is not enough because the product can show local errors not detected with them. To assess an orthophoto it is necessary to know the quality of the flight, the digital model surface and the mosaicking. Assessing all the stages in flowchart production we discover the origin of possible errors in the final product. The next paragraphs describe some check controls related with spatial resolution that are not considered in general mapping tests.

Referring to the flight, this check can be divided in geometric and radiometric controls. From a geometric point of view, it is necessary to evaluate if there are overlapped images on the whole area to guarantee stereoscopic vision. Moreover, a critical aspect is that the GSD of the flight planning agrees with the GSD of the flight. If GSD of the flight is higher, it will be possible to produce an orthophoto but not with the expected GSD. This control lets us know the accuracy of the navigation system of the UAV used. Taking into account the radiometric aspect, it is necessary to evaluate if the images are unfocused or distorted. In addition, depending on the application, it could be necessary to analyze image histograms, saturation, *etc.*

Taking into account the high density of points of DSM it can be possible to produce a true-orthophoto. This process requires manual editing to define breaklines, for example like in buildings. This process is necessary because orthophotos would show undefined borders in elevated objects, distortion in objects, *etc.* In this case the quality of DSM is critical to make a visual inspection to detect errors. In an urban scenario, it is necessary to consider it on the flowchart, not being critical in a rural scenario. Another solution could be to use a Digital Elevation Model, filtering the cloud point of DSM. In this case we would produce a classic orthophoto. In the study area, errors cause by problems with DSM where not important because of the type of study area. Problems with seamlines are possible to detect in mosaicking with visual inspection. Therefore, problems with the geometric and radiometric continuity are also located.

In this context, it is evident a sample of points is not enough to asses spatial quality. Standard mapping tests are focused only in coordinates, but it is necessary to check and report other aspects that also affect spatial quality of the product.

According to these results, NMAs can use UAVs in their flowchart of cartographic production, especially in urban maps (scales ranging from 1:500 to 1:2000) rather than in territorial maps (scales of 1:5000 and lower). This is an encouraging result because in the process of updating geographic information in urban maps, working areas can normally be covered by a UAV flight, considering the duration of batteries. In territorial maps, however, areas are larger and quite difficult to cover with multi-rotor UAV flights. Good temporal resolution is required in urban scenarios where the territory is highly dynamic because of human activity. UAVs can be a good alternative for updating geodatabases because of their facility of operation, low cost compared to manned flights and availability.

## 4. Conclusions

In this study we used five methods to assess the positional accuracy of an orthophoto obtained from images taken from a multi-rotor UAV. The high resolution and accuracy of the orthophoto required a careful planning of image acquisition. It was necessary to take into account the GSD of the flight and altitude above ground level and to measure ground control points to use in the aerial triangulation.

The tests used by NMAs to assess geomatic products that we applied in this study showed a broad range of valid scales for the UAV orthophoto, ranging from 1:250 to 1:1000, considering an orthophoto GSD of 0.05 m. Taking into account the human limit of visual perception and the GSD of these images, the most appropriate scale to generate the orthophoto was 1:1000. This scale is linked to an orthophoto GSD of 0.10 m. This is more than sufficient to support the error transmission in the flowchart of orthophoto production considering a flight GSD of 0.05 m. Other scales such as 1:500 were ruled out because the GSD of the flight was the same as the GSD of the orthophoto. A slight increase in the flight altitude would increase the GSD of the image, which would therefore be higher than that of the orthophoto, making it invalid for this scale.

The UAV orthophoto obtained positive results in the tests used by NMAs. This proves that UAV platforms can be used as an alternative by these NMAs particularly to update urban maps because of the high temporal resolution required by users and the scale of the product.

## Figures and Tables

**Table 1.** Summary of results for each spatial quality test.

**Test**	**Result Quality Test**
NMAS	1:250
EMAS	1:1000
ASLSM	1:500
NSSDA	0.144 m
STANAG	1:500 RATE A
